# Twenty-Five Years of Pathophysiology-Based Surgery of Slow-Transit Constipation: Outcomes After Segmental, Subtotal and Total Colectomy

**DOI:** 10.3390/jcm15124727

**Published:** 2026-06-18

**Authors:** Gennaro Melone, Paolo Luffarelli, Ludovico Carbone, Chiara Cascone, Natale Calomino, Marzio Angelo Zullo, Valter Ripetti

**Affiliations:** 1 General Surgery Unit, Fondazione Policlinico Universitario Campus Bio-Medico, Via Alvaro del Portillo 200, 00128 Rome, Italy; gennaro.melone@unicampus.it (G.M.); p.luffarelli@policlinicocampus.it (P.L.); c.cascone@unicampus.it (C.C.); m.zullo@policlinicocampus.it (M.A.Z.); v.ripetti@policlinicocampus.it (V.R.); 2 Proctology and Pelvic Floor Surgery Unit, Fondazione Policlinico Universitario Campus Bio-Medico, Via Alvaro del Portillo 200, 00128 Rome, Italy; 3 General Surgery Unit, Azienda Ospedaliero Universitaria Senese, Banchi di Sotto 55, 53100 Siena, Italy; natale.calomino@unisi.it

**Keywords:** constipation, colectomy, Wexner score, postoperative complications, quality of life, recurrence

## Abstract

**Background:** Idiopathic slow-transit constipation (STC) is a clinically significant event of chronic constipation. Total colectomy with ileorectal anastomosis is considered the standard surgical option for medically refractory STC but is associated with relevant morbidity and long-term functional impairment. This study aimed to evaluate safety and functional outcomes of partial colectomy (segmental or subtotal resection) as a potential alternative in patients with limited colonic involvement. **Methods:** A retrospective observational single-center study was conducted on patients with STC refractory to medical and rehabilitative treatment (1998–2021). Five-year follow-up data were collected. **Results:** On a cohort of 76 patients, 10 (13.2%) underwent total colectomy, 63 (82.9%) segmental, and 3 (3.9%) subtotal resections (left hemicolectomy). No perioperative mortality occurred. Overall, 30-day morbidity was 25.0%, with major complications observed in 12.1% after partial colectomy. Median hospital stay was three days longer after total colectomy. Constipation recurred in 20.3% of patients, exclusively after segmental resection, at a median follow-up of 7.7 years. Constipation severity significantly decreased postoperatively (*p* < 0.001), with the mean Wexner score improving from 21.5 to 6.1 (*p* < 0.001). Rates of diarrhea and fecal incontinence were comparable between segmental and total colectomy. Quality-of-life significantly improved in more than 75% of cases across all SF-36 domains. **Conclusions:** Segmental colectomy may be a safe and effective alternative to total colectomy in patients with limited STC, potentially offering durable symptom relief and favorable quality-of-life outcomes.

## 1. Introduction

Chronic constipation ranges between 3% and 27% in Western countries [1,2]. Although its etiology remains unclear, many predisposing factors have been identified including female gender, low socio-economic status, absence of fiber in the diet, and a reduced daily consumption of water and physical activity [3,4,5,6]. Hormonal and/or neuroendocrine mechanisms may partly explain the female predominance, including progesterone receptor overexpression and associated serotonergic pathway alterations reported in women with slow-transit constipation [7].

Differences in colonic transit and anorectal function allow chronic constipation to be classified into three subtypes: dyssynergic defecation, slow-transit constipation (STC), and normal-transit constipation [8]. STC is characterized by a delay in transit of stool through the colon, more frequent in women of childbearing age. Among patients with chronic constipation, 15 to 30% are estimated to have STC, which translates into an estimated prevalence of 2 to 4% in the general population. However, the severity of constipation is highly variable up to the complete cessation of spontaneous bowel movements, substantially limiting daily functioning and psychosocial well-being and, ultimately, compromising health-related quality of life [9,10].

Therefore, the management of STC is challenging in clinical practice. Surgery becomes a key option after exclusion of other correctable causes and failure of conservative and pharmacological therapy [11,12]. The standard procedure is total colectomy, with ileorectal anastomosis with a temporary diverting ileostomy when technically feasible or an end ileostomy in selected high-risk patients. On the other hand, segmental colectomy has not demonstrated superiority over total colectomy in the presence of diffuse colonic dysmotility. Nevertheless, total colectomy is associated with substantial morbidity. Pooled data indicate an overall complication rate of up to 24%, including 0.4% mortality, 13% reoperation, and 15% small-bowel obstruction. Long-term symptoms are also frequent, with abdominal pain reported in 30–50% of patients, bloating in 10–40%, recurrent constipation in 10–30%, and diarrhea or incontinence in 5–15% [13,14].

In the era of organ-sparing surgery, partial colectomy has been considered as an alternative approach in patients with limited colonic involvement by STC. The purpose of this study is to evaluate the perioperative outcomes and long-term functional results of partial colectomy and total colectomy for medically refractory idiopathic STC, within a pathophysiology-guided treatment framework.

## 2. Materials and Methods

### 2.1. Study Design, Setting and Ethics

We conducted a single-center retrospective observational study at the Fondazione Policlinico Universitario Campus Bio Medico (Rome, Italy). From 1998 to 2021, 1160 patients with chronic constipation were evaluated, and 76 adults (≥18 years) with STC refractory to optimized conservative therapy (medical, behavioral and rehabilitative) underwent surgery.

The inclusion criteria were a diagnosis of idiopathic STC confirmed by colonic transit study and failure of optimized conservative therapy sustained for a minimum of 12 months. Nutritional and behavioral therapy included a high-fiber diet (≥25 g/day), adequate hydration (≥1.5 L/day), the possible introduction of coffee, as caffeine stimulates the secretion of acetylcholine, regularized defecation timing, and physical activity counseling. Pharmacological therapy required the sequential failure of at least two laxative classes and, in recent years, prokinetic agents. Pelvic-floor rehabilitation consisted of at least 2 months of biofeedback-guided training and anorectal manometry-directed exercises. Psychological support included psychosocial assessment and, where indicated, cognitive–behavioral therapy or psychiatric evaluation to address comorbid anxiety or depression. Additional inclusion criteria were the availability of a standardized preoperative work-up as described, preoperative Wexner Constipation Score, and SF-36 questionnaire. Patients with obstructed defecation syndrome without STC, irritable bowel syndrome with constipation (IBS C), functional defecation disorder (pelvic floor dyssynergia) and secondary causes (organic, endocrine metabolic, neurologic/psychiatric, or drug induced) were excluded.

The results of this study were reported according to the “Strengthening the reporting of observational studies in epidemiology” (STROBE) statement for cohort studies (https://www.strobe-statement.org).

### 2.2. Preoperative Diagnostic Framework

STC was defined by delayed colonic transit assessed using the Metcalf technique. Patients ingested three sets of distinctive radiopaque markers administered over three consecutive days. A plain abdominal X-ray was obtained on day 4 to estimate colonic transit, based on the number and segmental distribution of retained markers; if markers were still present at day 4, an additional abdominal X-ray was performed on day 5 to confirm its delay. Localized colonic transit delay was defined as predominant marker retention confined to one colonic segment (mainly a sigmoid resection), with adequate progression or clearance in the remaining colon. Subtotal colectomy was selected in patients with predominant proximal or distal stasis not involving the entire colon. Diffuse colonic transit delay, or diffuse colonic inertia, was defined as marker retention distributed throughout the colon, involving all major colonic segments, or as a non-segmental pattern without a dominant site of retention. Anorectal manometry, defecography and colonoscopy were used to exclude outlet obstruction and structural disease before surgery.

### 2.3. Surgical Procedures

**Figure 1:**
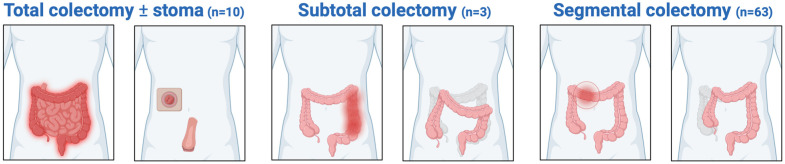
Total and partial (subtotal, segmental) colectomy.

Total colectomy with ileorectal anastomosis and diversion was indicated in diffuse colonic inertia. Segmental colectomy was defined as the resection of one colonic segment (mostly a sigmoid resection) based on the distribution of a localized delayed transit on marker studies, with restoration of continuity via primary colo-colic/colo-rectal anastomosis. Subtotal colectomy was defined as resection of the left or right part of the colon, performed in patients with predominant distal or proximal stasis not fulfilling criteria for total colectomy. Thus, segmental and subtotal colectomies were categorized as “partial”. (Figure 1) [15].

### 2.4. Follow-Up

Perioperative outcomes at 90 days were analyzed from all 76 available clinical records. The 30-day complications included fever, hypotension, delayed bowel function, wound infection, urinary retention, urinary tract infection, atrial fibrillation, anemia, rectal bleeding, anastomotic leakage, intra-abdominal hematoma, and anastomotic stenosis; while the 90-day complications encompassed sub-occlusion, bowel obstruction, perforation, and postoperative bowel syndrome.

**Figure 2:**
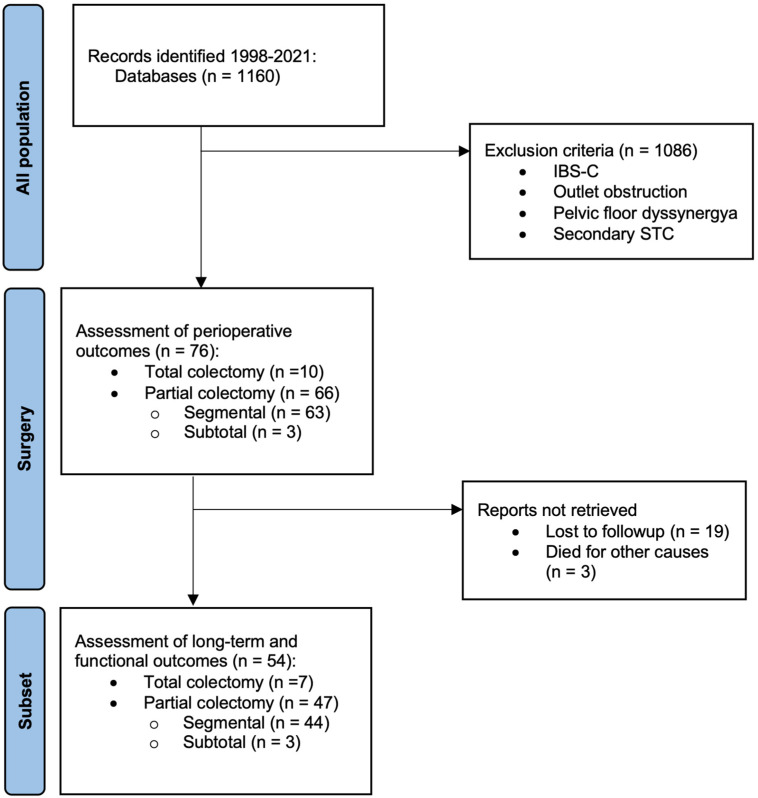
Flowchart.

Patients underwent follow-up every 6 months for 2 years and annually for an additional 3 years, unless symptoms recurred. Overall, 54 patients provided evaluable responses (19 were lost to follow-up, and 3 had died of unrelated causes) (Figure 2). The characteristics of these patients were compared with those of completers to assess potential systematic differences.

Laxative or enema dependence, evacuation frequency, difficulty and incompleteness of defecation, daily unsuccessful evacuation attempts, abdominal pain (defined as chronic, recurring abdominal pain or cramping reported by the patient), and diarrhea or fecal incontinence were registered.

Functional outcomes were assessed using the Wexner constipation score [16,17,18] and quality of life through the SF-36 domain scores [19,20,21]. SF-36 items were administered through a physician-administered telephone survey and collected in a dichotomous format reflecting the absence/presence of limitations or symptoms. Each item was recoded so that higher values consistently indicated better health status, and items were aggregated into the eight standard SF-36 domains (Physical functioning, Role-physical, Bodily pain, General health, Vitality, Social functioning, Role-emotional, and Mental health) according to the original item-to-domain structure.

Constipation recurrence was defined as the return of symptoms meeting the original inclusion criteria for STC, using the Wexner Constipation Score, associated with resumption of regular laxative or enema use or the evacuation frequency returning to preoperative levels.

### 2.5. Endpoints

The primary aim was a change in constipation severity, using the Wexner score, from baseline to last follow up among respondents [16,17,18]. The patient-reported quality of life (SF 36 domain scores) [19,20,21], stool frequency, laxative/enema dependence, diarrhea and fecal incontinence, 30- and 90-day complications (Clavien–Dindo, minor grade I–II and major grade III–IV), and recurrence of constipation (time to event) were secondary endpoints.

### 2.6. Statistical Analysis

Continuous variables are presented as the mean and standard deviation (SD) or median with interquartile (IQ) range and the categorical variables as n (%). Group comparisons used Fisher’s exact test for categorical data and appropriate non-parametric tests for continuous data. Effect sizes (mean differences or risk differences with 95% CIs) are presented where informative. Time to recurrence was analyzed by Kaplan–Meier with log rank test. Two-sided *p* < 0.05 was considered statistically significant. The study size was not calculated a priori, as all eligible patients (inclusion/exclusion criteria) treated during the study period were included. Analyses were performed with SPSS 26.0 software package (IBM Corporation, Armonk, NY, USA).

## 3. Results

The cohort of 76 patients fulfilling the inclusion criteria comprised 94.7% women, with a median age of 53 years and a median BMI of 23.6 kg/m^2^.

Most patients (82.9%) underwent segmental colonic resection, predominantly removing the sigmoid segment. Total colectomies were completed in 10 patients (13.2%), with left hemicolectomy in three (3.9%). (Table 1) The operation time was longer in the total colectomy (219 ± 42 min) vs. the segmental (192 ± 48) or subtotal (201 ± 7). The mean blood loss was 138 ± 75 mL in the total colectomy, 155 ± 195 mL in the segmental, and 100 ± 10 mL in the subtotal.

### 3.1. Short-Term Course

The median length of hospital stay was six days, and the overall postoperative complications rates are described in Table 2. Most minor (effect size 2.04; 95% Cl 0.82–5.00) and major (effect size 3.30; 95% Cl 0.33–33.13) complications occurred in the first 30 days after surgery.

Minor complications included the following: one case of fever and one case of delayed bowel transit after total colectomy; four cases of surgical site infections and one urinary tract infection, two bleedings, two cases of substenosis, two cases of cardiac arrythmia; one patient experienced delayed bowel transit after segmental colectomy. Severe postoperative complications occurred in 11 patients who underwent partial colectomy (30-day: two anastomotic leaks, one bleeding, one anastomotic stenosis and four bowel (sub)occlusion; 90-day: one bleeding and two bowel obstructions), treated with endoscopic intervention in five cases and radiological/percutaneous in two cases, while reoperation was needed in four patients. No unplanned intensive care-unit admission (ICU) or related-disease mortality was recorded. No differences in postoperative complications rate have been noted between open and laparoscopic approaches (30-day: minor *p* = 0.783, major *p* = 0.165; 90-day: minor *p* = 0.616, major *p* = 0.578).

### 3.2. Recurrence

The median follow-up in the subset group of 54 patients was 8.9 years (range 5.2–13.2) (Table 3). Other patients were female with a median age of 58 years and a median BMI of 23.2 kg/m^2^.

**Figure 3:**
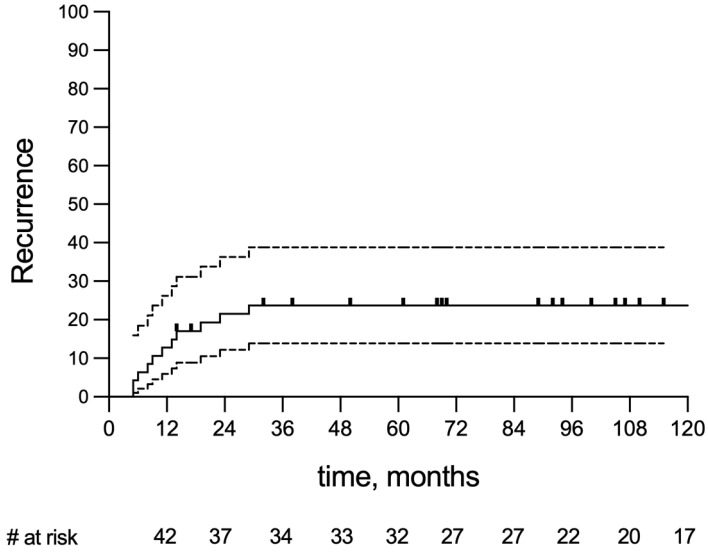
Recurrence probability after partial colectomy. Dashed lines indicate the 95% confidence interval; tick marks denote censored observations.

Evidence of chronic constipation after surgery was described in 11 (20.3%) cases of segmental resections occurring at a median time of 7.7 years (IQ range, 2.7–11.2). No cases of constipation recurrence occurred after subtotal colectomy (Figure 3). All patients presenting recurrent constipation were female with a median age of 56 years and a median BMI of 24.7 kg/m^2^. Within this subgroup, the following additional procedures were performed: three Longo hemorrhoidopexies, four stapled prolapsectomies, one proctopexy, two sacral neuromodulations, and one total colectomy.

### 3.3. Functional Outcomes

Table 4 reports the comparison of preoperative and postoperative functional outcomes. The laxative dependence (*p* < 0.001), use of evacuative enemas (*p* < 0.001) and abdominal pain (*p* < 0.001) decreased after surgery.

The number of monthly stool evacuations significantly increased after surgery. A total colectomy increased evacuations from 4.71 ± 2.36 to 34 ± 11.48 (*p* < 0.001), a segmental colectomy from 6.37 ± 6.55 to 24.20 ± 14.11 (*p* < 0.001), and a subtotal colectomy from 2.66 ± 1.15 to 25 ± 5 (*p* = 0.023).

**Figure 4:**
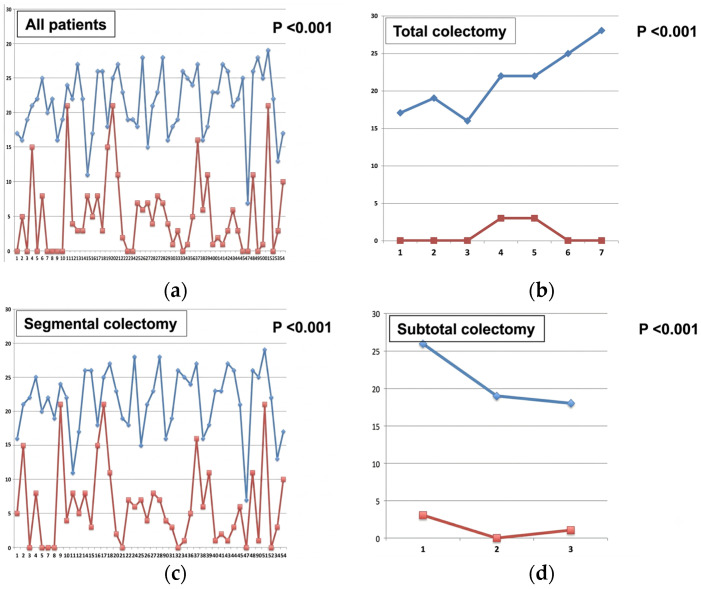
Comparison of preoperative (blue line) and postoperative (red line) Wexner score: (**a**) in all patients (N = 54); (**b**) in patients undergoing total colectomy resection (N = 7); (**c**) in patients undergoing segmental resection (N = 44); (**d**) in patients undergoing subtotal resection (N = 3).

The mean preoperative Wexner score in the entire population was 21.5 ± 4.7 (total colectomy 21.3 ± 4.3 vs. partial colectomy 21.5 ± 4.8) and decreased in the postoperative period to 5.2 ± 5.7 (total colectomy 0.8 ± 1.5 vs. partial colectomy 5.8 ± 5.9), even after stratification for surgical procedure (*p* < 0.001) (Figure 4).

### 3.4. Quality of Life

Improvements in SF-36 domain scores are reported in Table 5. Patients reported an increase in the rate of treatment success of 85.7%, 75 to 79.5% and 100% in all domains after total, segmental and subtotal colectomy, respectively.

## 4. Discussion

The surgical management of STC remains controversial, largely because delayed colonic transit frequently overlaps with defecatory disorders and outlet dysfunction, which can influence postoperative satisfaction and long-term function. Therefore, a pathophysiology-based preoperative work-up, including transit testing and dedicated anorectal and pelvic floor assessment, could be crucial to tailor the extent of resection [22].

In our experience, surgery for medically refractory STC was feasible, with no perioperative mortality and a shorter length of stay, ultimately resulting in meaningful improvements in constipation severity and evacuation frequency. These findings align with the broader literature showing that colectomy can benefit carefully selected STC patients, while emphasizing that postoperative outcomes remain heterogeneous.

A quantitative benchmark is provided by the group of researchers of the CapaCiTY systematic review, encompassing 40 studies, in which colectomy for STC was associated with a mean length of stay of 10.4 days (range 7.0–15.5) and a pooled overall complication rate of 24.4% (95% CI 17.8–31.7), with an early postoperative ileus and adhesional small-bowel obstruction (SBO) of 9.7% (95% CI 5.7–14.6). Importantly, although the mortality was low in that cohort, it was not negligible (0.4%, 6/1568) [23]. In our cohort, short-term outcomes were comparable in magnitude, with a shorter hospital stay and no perioperative mortality. Overall, the 30-day morbidity was 25.0% (14.5% minor and 10.5% major complications according to the CD classification), and no major complications were observed after total colectomy, albeit in a substantially limited number of patients, with different baseline characteristics, which does not allow for a comparative analysis. Nevertheless, non-invasive or surgical treatment of major complications did not result in unplanned intensive care-unit admission or mortality. Moreover, in patients at high risk of anastomotic leakage, new technique such as fluorescence-guided could allow for the identification of well-vascularized intestinal segments and further reduce the likelihood of surgical complications [24].

Beyond perioperative morbidities, the long-term “price” of colectomy deserves explicit discussion, especially because STC often affects young patients, for whom lifelong sequelae may have a disproportionate impact. In the same systematic review, the overall global satisfaction was high (85.6%, 95% CI 81.4–89.3), but persistent or de novo symptoms were common: diarrhea 9.8%, fecal incontinence 7.4%, recurrent/ongoing constipation 18.2%, persistent abdominal pain 39.3%, and bloating 23.9%. Recurrent SBO occurred in 15.2% (95% CI 10.2–20.9), and poor functional outcomes led to further resection or permanent stoma in a median 5% of patients (range 0–28%) [23]. Consistently, historical single-center series have long highlighted SBO and the need for reoperation after colectomy as clinically meaningful late burdens over decades of follow-up [25,26,27]. Selected reports also describe a non-trivial rate of subsequent procedures (e.g., adhesiolysis, anastomotic revision, or diversion in a minority), reinforcing the importance of shared decision-making when proposing total colectomy in this young population [28].

From a practical standpoint, total colectomy, with ileorectal anastomosis, if necessary, should remain the reference operation for diffuse colonic inertia, because it removes the entire dysfunctional colon and minimizes the risk of leaving behind dysmotile segments; contemporary series continue to report durable symptom improvement and patient benefit in appropriately selected cases [29,30,31]. In a focused series of 37 STC patients who underwent total colectomy, Sohn et al. reported no postoperative mortality and a marked improvement in constipation severity (Wexner 19.3 to 2.3, *p* < 0.001), with early complications in 13.5% and late complications in 18.9%, largely driven by obstruction/ileus; nevertheless, 81.8% of evaluable patients were satisfied, and the mean stool frequency was 3.6/day [31]. High-quality long-term data further support that, in strictly selected patients, a total colectomy can yield durable benefit with a reassuring quality of life: in the physiologically characterized Mayo cohort (110 patients; median follow-up 11 years), 98% reported improvement, and 85% were satisfied with bowel function; validated instruments showed substantial symptom improvement and SF-12 physical and mental summary scores comparable to the normal population, indicating that long-term quality of life can be preserved when the selection is rigorous, and the pelvic floor dysfunction is appropriately addressed [29].

At the same time, our findings support the concept that a small rigorously selected subgroup, namely patients with localized segmental delay documented by transit testing, may achieve comparable functional outcomes after colon-sparing resections. In our cohort, the symptom burden improved substantially after surgery with a marked reduction in constipation severity (mean Wexner 21.5 to 5.2, *p* < 0.001) and a significant increase in evacuation frequency across procedures; in the long-term follow-up subset (median 8.9 years), the quality of life improved across several SF-36 domains. Importantly, recurrence occurred in 20.3% of patients, exclusively after segmental resections at a median of 7.7 years and rarely required a total colectomy. Subsequent interventions were predominantly directed at obstructive components (e.g., hemorrhoidopexies, prolapse procedures, proctopexies, neuromodulation), suggesting that “failure” may often reflect a mixed constipation phenotype rather than inadequate resection. These data reinforce the clinical rationale for colon preservation, particularly in younger patients, because preserving the bowel length may mitigate downstream sequelae associated with extensive resection (e.g., high stool frequency, dehydration/electrolyte imbalance) and may reduce the lifelong risk of adhesional SBO/reoperations, while still delivering meaningful constipation relief in carefully selected localized disease.

This tailored approach aligns with the contemporary emphasis that only a minority of constipated patients meet the stringent criteria for colectomy and that careful selection is essential to maximize the benefit and minimize adverse outcomes [32]. The strengths of this study include a long institutional experience with a pathophysiology-guided selection protocol and a prolonged follow-up (median 8.9 years). Nevertheless, our study is limited by its retrospective design, the relatively small number of total/subtotal colectomies limiting comparisons, the inherent heterogeneity in STC phenotypes, and the selection bias to assigning total colectomy for diffuse versus partial colectomy for localized disease. Patients assigned to total colectomy presented with markedly longer symptom duration (median 30 vs. 12 years) and lower BMI (median 19.2 vs. 24.0 kg/m^2^), consistent with a more refractory and potentially malnourished phenotype. However, matching or similar balancing techniques at this stage were unfeasible, as their application would have further reduced an already small sample. Furthermore, the surgical approach, with laparoscopy used in 61.9% of cases, could represent an additional potential confounder that was not formally analyzed, as minimally invasive surgery has been independently associated with a reduced length of stay, lower wound complication rates, and faster functional recovery in other series. Notably, the SF-36 administration as a physician-delivered telephone survey with dichotomized responses deviates from the validated multi-level format, limiting comparability with other published series. As the assessors were not blinded to the surgical procedure performed, a degree of detection bias cannot be excluded. Finally, identifying predictive factors for successful partial colectomy (and for late recurrence) will require larger cohorts with standardized physiology testing and harmonized endpoints, enabling robust multivariable modeling without overfitting.

## 5. Conclusions

Total colectomy remains the standard surgical approach for patients with diffuse colonic inertia. In selected patients with localized colonic transit delay and appropriately evaluated outlet dysfunction, colon-preserving resections may represent a feasible surgical option worth investigating. Segmental colectomy may offer the potential benefit of bowel preservation, despite a potential risk of recurrence. Prospective studies with standardized selection criteria are needed to confirm these observations.

## Figures and Tables

**Table 1:** Patients’ characteristics.

Variables	All (N = 76)	Total (N = 10)	Partial (N = 66)	Segmental (N = 63)	Subtotal (N = 3)
Female gender	72 (94.7)	9 (90.0)	63 (95.5)	60 (95.2)	3 (100.0)
Age, years (range)	50.5 (21–77)	53.5 (24–67)	52 (44–66)	51 (19–87)	54 (43–62)
BMI, kg/m^2^ (range)	24.0 (18–33)	19.2 (18–25)	24 (22.0–28)	23.9 (18–33)	24.7 (20–31)
Symptoms’ duration before surgery, years (range)	18.5 (1–60)	30 (2–40)	12 (5–25)	11 (1–60)	15 (1–20)
Familiarity with chronic constipation	16 (21.1)	1 (10.0)	15 (22.7)	13 (20.6)	2 (66.6)
Laparoscopy	47 (61.9)	5 (50.0)	42 (63.6)	40 (63.5)	2 (66.6)

Acronym: BMI: Body Mass Index.

**Table 2:** Postoperative complications stratified by Clavien–Dindo classification (CD).

Variables	All (N = 76)	Total (N = 10)	Partial (N = 66)	*p*-Value
Hospital Stay, days (range)	6 (5–8)	9 (3–22)	6 (5–7)	0.087
30-day morbidity:				
• Minor (CD I–II)	11 (14.5)	2 (20.0)	9 (13.6)	0.594
• Major (CD III–IV)	8 (10.5)	0 (0)	8 (12.1)	0.541
90-day morbidity:				
• Minor (CD I–II)	4 (5.3)	0 (0)	4 (6.3)	0.968
• Major (CD III–IV)	3 (3.9)	0 (0)	3 (4.5)	0.854

**Table 3:** Characteristics of 54 patients who completed 5-year follow-up.

Variables	All (N = 54)	Total (N = 7)	Partial (N = 47)	Segmental (N = 44)	Subtotal (N = 3)
Female gender	50 (92.6)	6 (85.7)	44 (93.6)	41 (93.2)	3 (100)
Age, years (range)	50.5 (21–77)	41 (24–59)	49 (43–61.5)	45 (21–77)	58 (43–62)
BMI, kg/m^2^ (range)	24 (18–33)	19 (18–25)	24 (21–27)	21 (18–33)	26 (20–31)
Symptoms duration before surgery, years (range)	18.5 (1–60)	30 (5–40)	15 (10–25)	7.5 (5–60)	17.5 (1–20)
Familiarity with chronic constipation	16 (29.6)	1 (14.3)	15 (31.9)	13 (6.8)	2 (33.3)

Acronym: BMI: Body Mass Index.

**Table 4:** Functional results after total and partial resections.

Variables	Preoperative Period				Postoperative Period				*p*-Value ^1^
	All (N = 54)	Total (N = 7)	Partial (N = 47)	*p*-Value	All (N = 54)	Total (N = 7)	Partial (N = 47)	*p*-Value	
Laxative	49 (90.7)	7 (100)	44 (93.1)	0.491	12 (22.2)	0 (0)	12 (27.2)	0.130	<0.001
Enema	44 (81.5)	6 (85.7)	38 (79.5)	0.757	4 (7.4)	0 (0)	4 (8.5)	0.422	<0.001
Abdominal pain	48 (88.9)	6 (85.7)	42 (90.7)	0.755	12 (22.2)	2 (28.5)	10 (22.7)	0.665	<0.001
Diarrhea	-	-	-	-	14 (25.9)	2 (28.5)	12 (20.4)	0.864	-
Fecal incontinence	-	-	-	-	13 (24.1)	1 (14.2)	8 (17.0)	0.856	-

^1^ Differences between preoperative and postoperative periods.

**Table 5:** Number of patients reporting improvement in each SF-36 domain at last follow-up, by surgical procedure.

Variables	All (N = 54)	Total (N = 7)	Partial (N = 44)	Subtotal (N = 3)
Social functioning	43 (79.6%)	6 (85.7%)	34 (77.3%)	3 (100%)
Physical functioning	44 (81.5%)	6 (85.7%)	35 (79.5%)	3 (100%)
Role emotional	43 (79.6%)	6 (85.7%)	34 (77.3%)	3 (100%)
Mental health	43 (79.6%)	6 (85.7%)	34 (77.3%)	3 (100%)
General health	43 (79.6%)	6 (85.7%)	34 (77.3%)	3 (100%)
Vitality	44 (81.5%)	6 (85.7%)	35 (79.5%)	3 (100%)
Pain reduction	42 (77.8%)	6 (85.7%)	33 (75.0%)	3 (100%)
Satisfaction	44 (81.5%)	6 (85.7%)	35 (79.5%)	3 (100%)

## Data Availability

The data presented in this study are available on request from the corresponding author due to privacy restrictions.

## Abbreviations

The following abbreviations are used in this manuscript:

STC	Slow-Transit Constipation
CD	Clavien–Dindo classification
BMI	Body Mass Index
IBS C	Irritable Bowel Syndrome with Constipation
SBO	Small-Bowel Obstruction

## References

[1] Pare P., Ferrazzi S., Thompson W.G., Irvine E.J., Rance L. (2001). An Epidemiological Survey of Constipation in Canada: Definitions, Rates, Demographics, and Predictors of Health Care Seeking. Am. J. Gastroenterol..

[2] Suares N.C., Ford A.C. (2011). Prevalence of, and Risk Factors for, Chronic Idiopathic Constipation in the Community: Systematic Review and Meta-Analysis. Am. J. Gastroenterol..

[3] Cullen G., O’Donoghue D. (2007). Constipation and Pregnancy. Best Pract. Res. Clin. Gastroenterol..

[4] Chan A.O.O., Lam K.F., Hui W.M., Leung G., Wong N.Y.H., Lam S.K., Wong B.C.Y. (2007). Influence of Positive Family History on Clinical Characteristics of Functional Constipation. Clin. Gastroenterol. Hepatol..

[5] Dukas L., Willett W.C., Giovannucci E.L. (2003). Association between Physical Activity, Fiber Intake, and Other Lifestyle Variables and Constipation in a Study of Women. Am. J. Gastroenterol..

[6] Adibi P., Abdoli M., Daghaghzadeh H., Keshteli A.H., Afshar H., Roohafza H., Esmaillzadeh A., Feizi A. (2022). Relationship between Depression and Constipation: Results from a Large Cross-Sectional Study in Adults. Korean J. Gastroenterol..

[7] Guarino M., Cheng L., Cicala M., Ripetti V., Biancani P., Behar J. (2011). Progesterone Receptors and Serotonin Levels in Colon Epithelial Cells from Females with Slow Transit Constipation. Neurogastroenterol. Motil..

[8] Black C.J., Ford A.C. (2018). Chronic Idiopathic Constipation in Adults: Epidemiology, Pathophysiology, Diagnosis and Clinical Management. Med. J. Aust..

[9] Serra J., Pohl D., Azpiroz F., Chiarioni G., Ducrotté P., Gourcerol G., Hungin A.P.S., Layer P., Mendive J., Pfeifer J. (2020). European Society of Neurogastroenterology and Motility Guidelines on Functional Constipation in Adults. Neurogastroenterol. Motil..

[10] Johanson J.F., Kralstein J. (2007). Chronic Constipation: A Survey of the Patient Perspective. Aliment. Pharmacol. Ther..

[11] Arebi N., Kalli T., Howson W., Clark S., Norton C. (2011). Systematic Review of Abdominal Surgery for Chronic Idiopathic Constipation. Color. Dis..

[12] Wong S.W., Lubowski D.Z. (2007). Slow-Transit Constipation: Evaluation and Treatment. ANZ J. Surg..

[13] Lundin E., Karlbom U., Påhlman L., Graf W. (2002). Outcome of Segmental Colonic Resection for Slow-Transit Constipation. Br. J. Surg..

[14] Di Fabio F. (2010). Poor Quality of Life in Patients Undergoing Total Colectomy and Ileorectal Anastomosis for Intractable Slow-Transit Constipation. Dis. Colon Rectum.

[15] Ripetti V., Caputo D., Greco S., Alloni R., Coppola R. (2006). Is Total Colectomy the Right Choice in Intractable Slow-Transit Constipation?. Surgery.

[16] Wexner S.D. (2021). Further Validation of the Wexner Incontinence Score: A Note of Appreciation and Gratitude. Surgery.

[17] Jorge M.J.N., Wexner S.D. (1993). Etiology and Management of Fecal Incontinence. Dis. Colon Rectum.

[18] Vaizey C.J., Carapeti E., Cahill J.A., Kamm M.A. (1999). Prospective Comparison of Faecal Incontinence Grading Systems. Gut.

[19] Lins L., Carvalho F.M. (2016). SF-36 Total Score as a Single Measure of Health-Related Quality of Life: Scoping Review. SAGE Open Med..

[20] Apolone G., Mosconi P. (1998). The Italian SF-36 Health Survey. J. Clin. Epidemiol..

[21] Ware J.E. (2000). SF-36 Health Survey Update. Spine.

[22] Bharucha A.E., Lacy B.E. (2020). Mechanisms, Evaluation, and Management of Chronic Constipation. Gastroenterology.

[23] Knowles C.H., Grossi U., Chapman M., Mason J. (2017). Surgery for Constipation: Systematic Review and Practice Recommendations. Color. Dis..

[24] Roviello F., Andreucci E., Carbone L., Calomino N., Piccioni S., Bobbio L., Piagnerelli R., Fontani A., Marrelli D. (2025). Preoperative Injection of Indocyanine Green Fluorescence at the Anorectal Junction Safely Identifies the Inferior Mesenteric Artery in a Prospective Case-Series Analysis of Colorectal Cancer Patients. Gastrointest. Disord..

[25] Lubowski D.Z., Chen F.C., Kennedy M.L., King D.W. (1996). Results of Colectomy for Severe Slow Transit Constipation. Dis. Colon Rectum.

[26] Nyam D.C.N.K., Pemberton J.H., Ilstrup D.M., Rath D.M. (1997). Long-Term Results of Surgery for Chronic Constipation. Dis. Colon Rectum.

[27] Webster C., Dayton M. (2001). Results after Colectomy for Colonic Inertia: A Sixteen-Year Experience. Am. J. Surg..

[28] FitzHarris G.P., Garcia-Aguilar J., Parker S.C., Bullard K.M., Madoff R.D., Goldberg S.M., Lowry A. (2003). Quality of Life After Subtotal Colectomy for Slow-Transit Constipation. Dis. Colon Rectum.

[29] Hassan I., Pemberton J.H., Young-Fadok T.M., You Y.N., Drelichman E.R., Rath-Harvey D., Schleck C.D., Larson D.R. (2006). Ileorectal Anastomosis for Slow Transit Constipation: Long-Term Functional and Quality of Life Results. J. Gastrointest. Surg..

[30] Johnston B.J., Clark D.A., Warwick A.M. (2023). Long-term Outcomes of Total Colectomy for Severe Constipation. Color. Dis..

[31] Sohn G., Yu C.S., Kim C.W., Kwak J.Y., Jang T.Y., Kim K.H., Yang S.S., Yoon Y.S., Lim S.-B., Kim J.C. (2011). Surgical Outcomes after Total Colectomy with Ileorectal Anastomosis in Patients with Medically Intractable Slow Transit Constipation. J. Korean Soc. Coloproctol..

[32] Chaichanavichkij P., Vollebregt P.F., Tee S.Z.Y., Scott S.M., Knowles C.H. (2021). Slow-Transit Constipation and Criteria for Colectomy: A Cross-Sectional Study of 1568 Patients. BJS Open.

